# Effectiveness of exercise at workplace in physical fitness: uncontrolled randomized study

**DOI:** 10.1590/S1679-45082014AO2956

**Published:** 2014

**Authors:** Antônio José Grande, Valter Silva, Sérgio Alencar Parra

**Affiliations:** 1 Universidade Federal de São Paulo, São Paulo, SP, Brazil; 2 Universidade Estadual de Londrina, Londrina, PR, Brazil

**Keywords:** Physical fitness, Anthropometrics, Motor activity, Work, Occupational health

## Abstract

**Objective:**

To investigate the effectiveness of workplace exercise for employee health by means of health-related physical activity components.

**Methods:**

A randomized uncontrolled study with 20 workers was carried out during three months to evaluate a workplace exercise program. The selected outcomes were flexibility, body mass, fat percentage, lean mass, blood pressure, and heart rate. For statistical analysis, the paired *t* test and the intent-to-treat analysis were used.

**Results:**

There was a significant increase in weight, fat percentage, blood pressure, and heart rate. However the clinical significance was 10% in the size of the effect.

**Conclusion:**

The changes verified in the outcomes analyzed were not significant; the variables are within normality ranges proposed by academic organizations

## RESUMO

**Objetivo:**

Investigar a efetividade da ginástica laboral para a saúde dos trabalhadores por meio de componentes da aptidão física relacionados à saúde.

**Métodos:**

Estudo randomizado não controlado com 20 trabalhadores realizado durante três meses para avaliar um programa de ginástica laboral. Os desfechos selecionados foram: flexibilidade, massa corporal, percentual de gordura, massa magra, pressão arterial e frequência cardíaca. Para análise estatística, foram utilizados o teste *t* pareado e a análise de intenção de tratar.

**Resultados:**

Houve aumento significante no peso, percentual de gordura, pressão arterial e frequência cardíaca. Contudo, o significado clínico foi de 10% no tamanho do efeito.

**Conclusão:**

As alterações verificadas nos desfechos analisados não foram significativas as variáveis encontram-se dentro da normalidade propostas por instituições acadêmicas.

## INTRODUCTION

Workplace exercise (WE) is an intervention with specific physical exercises for workers carried out at the work site, which aims to improve general outcomes, such as Quality of Life and occupational environment, as well as specific outcomes, such as muscle strength and flexibility.^(1-7)^

Analyses of general outcomes of the workers involve ample constructs, and are generally evaluated by questionnaires. In this way, they depend on the individual perception of each worker, which can overestimate the effects of the intervention to the detriment of permanence in the health promotion program of the company.^(8,9)^

On the other hand, specific outcomes, such as strength, flexibility, and blood pressure may be quantified with measuring instruments, thus diminishing the bias of the workers' perception subjectivity and contributing towards the true measure of the intervention.^(10-14)^

Both types of outcomes are important and each one can be analyzed by different perspectives, whether quantitative (to measure the effect of the intervention, for example) or qualitative (to understand some aspects, such as barriers/facilitators, among others). The development of the study depends on what the research intends to investigate.

Cochrane's systematic reviews, which included controlled and randomized studies with exercises at the work place, observed the direction of the effect in favor of general outcomes, such as Quality of Life, but there were no significant effects in specific outcomes as to physical fitness.^(15,16)^

It is important to explain that exercises at the worksite vary in intensity, frequency, and volume among countries; for example, in Japan, repetition exercises are applied before the beginning of work for periods of 10 to 15 minutes. Whereas randomized and controlled studies in the United States analyze exercises with greater intensity and longer duration, on average, for 30 minutes per session. In Brazil, WE can be applied during the beginning, middle, or end of the work shift, generally with light intensity and duration of 10 to 15 minutes. These particularities of countries make WE challenging to researchers.^(17)^

The benefits of WE are not yet elucidated in the literature, and the methodological quality of the studies is still poor.^(15,16)^ However, the importance of physical exercise is already well documented and has a positive impact on physical fitness related to health and on the metabolic profile.^(18,19)^

In general, workers with a registered employment record card have a daily work load of 8 hours, that is, one third of the hours of a day, in which another third is spent sleeping, and the remaining third with transportation and daily activities, such as meals, leisure, and daily tasks.^(20)^

Some factors, such as lack of time and the heavy daily work load, contribute towards sedentarism. Therefore, the practice of exercises at the workplace has the potential of developing better habits of life, health indicators, and even benefit the country's public health.^(21)^

## OBJECTIVE

To investigate the effectiveness of workplace exercise for the health of workers by means of specific outcomes, that is, physical fitness components related to health.

## METHODS

### Type of study

This was a prospective, randomized, uncontrolled study, classified as a quasi-experimental research, since a single group of workers received the intervention of “physical exercise” at the workplace for a period of three months.

### Participants

Workers of a company in the administrative sector of the city of Londrina (PR) participated. The company was selected due to convenience, and had not previously participated in any employee health promotion program or received WE interventions in its history.

The company had 160 worked with signed registered employee cards, and 44-hour/week workloads. To establish the sample, meetings were held with the company's Human Resources Department. Thus, the company agreed with the researcher in freeing 20 workers to participate in WE.

To select who would be evaluated, a spreadsheet was drawn up attributing a number to each worker's name. Next, a sequence of 20 random numbers was generated, for a universe of the 160 possible. Randomization was made at the Research Randomizer site, version 4.0.^(22)^ This free site was developed to help students and researchers generate random numbers for research.

All participating employees read and signed the Informed Consent Form. The research procedures followed the guidelines given by Resolution 196/96 of the National Health Council and the research project was approved by the Ethics and Research Committee of the Methodist University of Piracicaba (UNIMEP) under protocol number 14/10.

### Inclusion criteria

The employees were to participate in the interventions at least twice a week. The frequency of participation was controlled by a list on which each employee signed his/her name.

### Intervention

Three WE sessions were offered, lasting 15 minutes per week, for three months. The sessions were composed of stretching exercises done individually and in pairs, with rods, with a ball, with latex tubes, and on the mat.

All the sessions were varied and diversified with the intention of maintaining participant compliance with the intervention. Chart 1 shows the 12 exercise sessions in detail.

**Chart 1:** Protocol of workplace exercises

Sessions of workplace exercise	Exercises proposed by body regions
Session 1	5 exercises of the upper limbs (4 minutes)
General familiarization and observation of the employees performing physical exercises, without materials	5 exercises of the lower limbs (4 minutes)
Static exercises	5 trunk exercises(4 minutes)
	3 cervical exercises (3 minutes)
Session 2	11 exercises of the upper limbs (8 minutes)
Emphasis on upper limbs, trunk, and cervical region, without materials	4 cervical exercises (2 minutes)
Static exercises	6 trunk exercises (5 minutes)
Session 3	11 exercises of the lower limbs (8 minutes)
Emphasis on the lower limbs, trunk, and cervical region, without materials	4 cervical exercises (2 minutes)
Static exercises	6 trunk exercises (5 minutes)
Session 4	5 exercises of the upper limbs (5 minutes)
Dynamic and static exercises for upper and lower limbs	5 exercises of the lower limbs (5 minutes)
	5 trunk exercises (3 minutes)
	3 cervical exercises (2 minutes)
Session 5	1 compression exercise with deep tissue gliding massage per region of the back
Massage with tennis balls, with emphasis on the back (relaxation)	(15 minutes free)
Session 6	8 exercises of the upper limbs (6 minutes)
Physical exercises with a rod, with emphasis on upper limbs	4 exercises of the lower limbs (3 minutes)
Dynamic and static exercises	4 trunk exercises (3 minutes)
	2 massage exercises (3 minutes)
Session 7	8 exercises of the lower limbs (7 minutes)
Physical exercises on the mat, with emphasis on lower limbs	4 trunk exercises (3 minutes))
Dynamic and static exercises	4 exercises of the upper limbs (3 minutes)
	2 exercises of the neck (2 minutes)
Session 8	5 exercises of the lower limbs (4 minutes)
Exercises sitting in a chair	5 trunk exercises (4 minutes)
Specific movements for the office position	3 cervical exercises (3 minutes)
	5 exercises of the upper limbs (4 minutes)
Session 9	6 exercises of the upper limbs (8 minutes)
Physical exercises with latex tubes	2 trunk exercises (2 minutes)
Resistance exercises for upper and lower limbs	3 exercises of the lower limbs (4 minutes)
Session 10	5 exercises of the upper limbs (6 minutes)
Physical exercises in pairs	4 trunk exercises (5 minutes)
	3 exercises of the lower limbs (4 minutes)
Session 11	5 exercises of the lower limbs (7 minutes)
Relaxation on the mat	5 trunk exercises (7 minutes)
Session 12	5 exercises of the upper limbs (5 minutes)
Physical exercises using the wall	5 trunk exercises (5 minutes)
Physical exercises supported on the wall	5 exercises of the lower limbs (5 minutes)

### Evaluation of outcomes

Evaluations were made during the morning period. Each participant was taken from his/her sector to a company room where the researcher made measurements. For the body composition analysis, a doubly segmented validated bioimpedance scale was used,^(14)^ (OMRON Sensing tomorrow TM, HBF500 model, Kyoto, Japan). Additionally, a validated electronic blood pressure measuring device was used (OMRON Sensing tomorrow TM, HEM-780 model, Kyoto, Japan),^(23)^ which simultaneously measured heart rate. Flexibility was assessed by a Chattanooga Group Baseline validated inclinometer.^(24)^

On the day of the initial evaluation, each employee went to the company room where the researcher made the evaluations. As per the protocol, the researcher asked the individual to sit down and remain at rest for 5 minutes. Next, the three blood pressure measurements were made, and the mean reading was used.

The second evaluation was focused on body composition, in which the employee took off his/her shoes and metallic elements on the body. The height used on the bioimpedance scales was stated by the employee. Next, he/she was asked to position both feet on top of the electrodes and to extend the arms forming a 90° with the trunk. The participant was asked that the palms of his/her hands and fingers firmly hold the upper limb electrodes.

With the third evaluation, flexibility was assessed in two motor actions. In the first, the participant was asked to lie in supine position on the mat; the researcher would flex the hip and place the inclinometer close to the greater trochanter of the femur. In the second, the employee would remain standing, with feet together, and would flex the trunk. The inclinometer was placed on the spinous process of the lumbar vertebra (L3). The acceptable measurements for the general population are between 90° and 170°,^(25)^ respectively.

### Statistical analysis

Per protocol analyses (PPA) were performed to evaluate the effect of the treatment and intent-to-treat (ITT) analyses were made to evaluate biases caused by loss of the segment. PPA included only the employees who completed the study. From the ITT analysis, the employees lost in follow-up had the value of each outcome of the initial evaluation attributed in the final evaluation.

Data tabulation and analysis were performed using the Statistical Package for Social Sciences software, version 18. Mean, standard deviation, and Student's *t* test for paired samples were used to compare the initial and final values. The level of significance adopted was p≤0.05.

## RESULTS

Twenty employees participated in the research project; five of them were male, with a mean age of 25.5±5.46 years, and 15 were female, with a mean age of 29.14±7.33 years. The outcomes researched are shown on table 1, which also contains the results of the initial evaluation, final evaluation, change from beginning, and level of significance, by analysis (ITT and PPA).

**Table 1:** Initial and final evaluations of the variables studied

		Initial evaluation	Final evaluation	Change from the beginning	p value
Body mass (kg)	ITT	72.48±18.49	73.11±19.18	0.64±2.44	0.2593
	APP	72.13±18.90	73.64±19.33	1.51±1.58	0.0024*
BMI (kg/m^2^)	ITT	25.04±5.26	26.00±5.19	0.96±2.88	0.1527
	APP	24.38±5.24	25.66±5.26	1.28±3.29	0.1542
Fat percentage (%)	ITT	31.61±8.51	32.97±8.02	1.36±2.40	0.0206*
	APP	30.78±9.53	32.58±907	1.80±2.63	0.0188*
Muscle mass (kg)	ITT	30.30±5.13	28.84±5.33	-1.46±3.32	0.0645
	APP	30.65±5.89	28.70±6.15	-1.94±3.72	0.0636
Flexibility of the hip (°)	ITT	81.50±20.27	81.55±9.50	0.25±4.13	0.7894
	APP	83.67 ±9.90	80.66±10.66	-3.00±7.51	0.1441
Lumbar flexibility (°)	ITT	163.25±14.89	165.25±15.26	2.00±5.23	0.1036
	APP	163.67±15.75	164.00±16.71	0.33±7.89	0.8736
RHR(bpm.min-1)	ITT	71.75±7.94	77.80±10.57	5.95±8.69	0.0064*
	APP	71.33±6.53	79.26±10.03	7.93±9.25	0.0051*
SAP (mm/Hg)	ITT	112.70±8.13	120.65±11.33	7.95±11.76	0.0070*
	APP	110.20±5.69	120.80±11.98	10.60±12.50	0.0056*
DAP (mm/Hg)	ITT	72.00±5.90	80.50±7.43	8.50±8.52	0.0003*
	APP	71.27±5.49	80.13±9.35	8.87±8.53	0.0001*

* Significant statistical differences p≤0.05.

ITT: intent-to-treat analysis; PPA: per protocol analysis; BMI: body mass index; RHR: resting heart rate; SAP: systolic arterial pressure; DAP: diastolic arterial pressure.

When the data was assessed by PPA, there was a significant increase in body mass, fat percentage, arterial pressure, and heart rate. The clinical significance, however, was small–about 10% in the size of the effect.

In the ITT analysis, with the exception of body mass, which showed no significant differences, all the variables showed behaviors similar to the PPA, including those non-significant.

In the final evaluation, there was loss of the segment of five women (three gave up the intervention and two were fired by the company). However, based on the ITT and PPA analyses, the sample loss did not reflect large differences by one method or the other, with exception for body mass. Thus, despite losses in follow-up, the size of the effect observed was not affected, reflecting the reality for the sample and the absence of bias due to the losses.

## DISCUSSION

Planning for this study occurred due to the need to launch new hypotheses in the area of occupational health related to WE, which has been used as a worker health-promoting intervention in various locations, such as hospitals, mines, universities, and companies.^(1-13)^ Many studies have found a significant and beneficial effect of this intervention, but the outlines of these studies do not allow generalizations.^(3,4,6-13)^

In this regard, this study allowed observation of the behavior of the outcomes studied after three months of intervention with physical exercise at the workplace, where each employee has his/her measurement compared at the initial and final moments of the intervention period.

Most workers of this company were female (approximately 70%), which justifies the fact that most of the randomized sample is female (75%). Among the outcomes studied, significant differences are noted between the initial and final evaluations of body mass (p=0.002), in fat percentage (p=0.019), in resting heart rate (p=0.005), in systolic arterial pressure (p=0.006), and in diastolic arterial pressure (p=0.001). There was risk of bias due to loss in follow-up regarding body mass. Despite the statistical significance, these values have little clinical importance, since all were within normative values. Additionally, the size of the effect of change from the beginning was approximately 10%, regardless of analysis by ITT or PPA.

Studies that researched the effects of WE on flexibility, body mass, and muscle force have found benefits.^(3,8-11)^ However, all the studies published in Brazil until now involving this intervention in the workplace had a high risk of bias.

A randomized and controlled study analyzing the WE and interventions of health education identified benefits of these interventions in the workplace, that is, the employees improved the social environment of the company.^(7)^

Despite the size of the sample, this study' strong point was the methodological care of randomizing the participants, thus eliminating the volunteer bias. The 25% sample loss is high, considering the small number of participants, but it should be noted that it was proportional for the men (1/5) and women (4/15), and can be attributed to the randomization. Furthermore, the results seem to not have been affected by the loss in follow-up, as was demonstrated in comparing the results of the ITT analyses, such as PPA.

It is important to highlight that, since it is a private company, the performance and participation in the research were seen as a period in which the employee was not producing, and therefore, the company lost profit. Therefore, the employer, generally, does not support this type of intervention since he/she does not accept its effect, increasing the chance of workers showing low compliance to the program.^(4)^

In this regard, research with WE has been developed in Brazil in partnerships with the Industrial Social Services (SESI) and public university workers by means of extension programs. Compliance with WE programs varies between 40%and 50% of the total number of employees.^(6,7,20,21)^

In a cross-sectional investigation comparing behaviors related to health among WE participants and non-participants in university workers, a greater consumption of alcohol and lower level of physical activity for leisure have been observed among non-participants. Thus, the hypothesis may be made that people who are already healthy seek such workplace exercise programs, which do not attract those who need to change their life styles.

One of the motives that might justify the current finding is the seasonality by the environmental temperature due to the different seasons of the year.^(26)^ This attribution came about by the fact of the research having been done between the months of June, July, and August, that is, during autumn and winter. Similarly, during these seasons, there is a tendency to gain weight by increased calorie intake and increased sedentarism, due to reduced physical activity as a possible explanation for the negative differences.

In another recently published clinical trial randomized by cluster, it was noted that intervention with WE significantly improves the occupational environment, *i.e.*, employees use this time to interact with their work colleagues, thus improving the social environment.^(7)^

Measuring the effects of WE in specific outcomes is difficult, due to the characteristics of the intervention, that is, 15-minute exercise sessions, of light intensity and, in most cases, carried out 3 times week. Therefore, we suggest that future research increase the intensity of effort, time of exercise session, and weekly frequency, which would increase the likelihood of identifying physiological differences.^(2)^

Although the suggestion above is obvious, a few problems may occur with compliance to more intense exercises, and the simple fact of returning to work with a sweaty body. These are two hypotheses that can be considered. Changing research to a qualitative view and employee satisfaction, WE justifies its existence, and may even contribute towards changes in behavior for an active lifestyle.

Workplace exercise is a strategic and new intervention with possibilities of growth due to the work routine. The acquisition of 10 to 15 minutes in the work routine should be extended and used for other purposes, such as health education.

Other research should explore interventions at work, with a greater number of workers, using a control group, and exploring several interventions in lifestyle.

## CONCLUSIONS

In this study, workplace exercise was not effective for improving outcomes of health-related physical fitness, with worsening of a few indicators (weight, fat percentage, heart rate, systolic arterial pressure, and diastolic arterial pressure). However, these results should be interpreted with caution, since they remain within population normative tables and the clinical significance was small – about 10% in the size of the effect.
